# LINE-1 silencing by retinoblastoma proteins is effected through the nucleosomal and remodeling deacetylase multiprotein complex

**DOI:** 10.1186/s12885-016-2068-9

**Published:** 2016-01-25

**Authors:** Diego E. Montoya-Durango, Kenneth A. Ramos, Pasano Bojang, Lorell Ruiz, Irma N. Ramos, Kenneth S. Ramos

**Affiliations:** Department of Biochemistry and Molecular Biology, University of Louisville School of Medicine, Louisville, KY 40202 USA; Department of Medicine, University of Arizona College of Medicine, Tucson, AZ 85721 USA; Department of Health Promotion Sciences, University of Arizona College of Public Health, Tucson, AZ 85721 USA

**Keywords:** Chromatin, Gene silencing, Long interspersed nuclear element-1, Retinoblastoma proteins, Nucleosomal and remodeling deacetylase complex

## Abstract

**Background:**

Long Interspersed Nuclear Element-1 (L1) is an oncogenic mammalian retroelement silenced early in development via tightly controlled epigenetic mechanisms. We have previously shown that the regulatory region of human and murine L1s interact with retinoblastoma (RB) proteins to effect retroelement silencing. The present studies were conducted to identify the corepressor complex responsible for RB-mediated silencing of L1.

**Methods:**

Chromatin immunoprecipitation and silencing RNA technology were used to identify the repressor complex that silences L1 in human and murine cells.

**Results:**

Components of the Nucleosomal and Remodeling Deacetylase (NuRD) multiprotein complex specifically enriched the L1 5′-untranslated DNA sequence in human and murine cells. Genetic ablation of RB proteins in murine cells destabilized interactions within the NuRD macromolecular complex and mediated nuclear rearrangement of Mi2-β, an ATP-dependent helicase subunit with nucleosome remodeling activity. Depletion of Mi2-β, RbAP46 and HDAC2 reduced the repressor activity of the NuRD complex and reactivated a synthetic L1 reporter in human cells. Epigenetic reactivation of L1 in RB-null cells by DNA damage was markedly enhanced compared to wild type cells.

**Conclusions:**

RB proteins stabilize interactions of the NuRD corepressor complex within the L1 promoter to effect L1 silencing. L1 retroelements may serve as a scaffold on which RB builds heterochromatic regions that regulate chromatin function.

## Background

Human L1s are non-LTR mammalian retrotransposons that consist of an internal bidirectional promoter, two open reading frames encoding for ORF1p (RNA-binding protein) and ORF2p (reverse transcriptase and endonuclease activities), a 3′- untranslated region (UTR), and a polyA tail [1]. Murine L1s share similar structural and functional features, except that the 5′-UTR is organized into monomeric units that function as an upstream promoter [2]. While L1s are highly active during early embryonic development, they are targeted for epigenetic silencing via DNA methylation and histone covalent modifications during the course of differentiation [3]. L1 reactivation is strongly associated with the acquisition of oncogenic phenotypes resulting from insertion mutagenesis and/or reprogramming of gene expression [1].

We have previously shown that recruitment of E2F/RB (E2F-retinoblastoma) to the L1 promoter, along with histone deacetylases 1 and 2 (HDAC1 and HDAC2, respectively) [4, 5] and methyl binding protein 2 (MBD2) [5] are critical to L1 silencing. Mouse embryo fibroblasts (MEFs) lacking all RB family members (pRb, p107, and p130) and referred to as triple knockout (TKO) MEFs, show impaired recruitment of HDACs to the L1 promoter and markedly enhanced L1 expression compared to wild type MEFs. Methyl CpG binding protein 2 (MeCP2) represses the transcriptional activity of L1 in transient reporter assays [6] and in vivo [7], and this activity is related to HDAC recruitment to methylated DNA [8]. The identity of the repressor complex that silences L1 has not yet been elucidated.

## Methods

### Cell models

MCF7 cells, HeLa cells, primary mouse embryo fibroblasts (MEFs) and RB null MEFs lacking all three family members (RB, p105 and p107) (TKO) were cultured in Dulbecco’s Modified Eagle’s medium (MultiCel; Cytosystems Pty. Ltd., Castle Hill, NSW, Australia) supplemented with 10 % fetal calf serum (FCS), 200 mg/ml streptomycin, and 200 U/ml penicillin G at 5 % CO2 and humidified air at 37 °C.

### Quantitative real time PCR

Total RNA was extracted and 1 μg used for cDNA synthesis (Invitrogen Superscript II). For each reaction, 25 μL of 2X SYBR green (Biorad) was mixed with forward and reverse primers to give a final concentration of 10 μm. One μL of cDNA mixture was brought up to 50 μL using DEPC water. Cycling conditions included an initial denaturation at 95 °C for 3 min followed by 50 cycles at 95 °C for 30 s, 55 °C for 30 s and 72 °C for 45 s. The homogeneity of PCR products was confirmed using a real time PCR melting curve.

### Chromatin immunoprecipitation

Cells were grown in 150 mm petri dishes to 90 % confluence, fixed in 1 % formaldehyde for 7 min and rinsed 3X with cold PBS. Chromatin was sheared to fragments below 1 Kb and 20 μg immunoprecipitated using selected antibodies. After purification, 0.5 to 2.0 μL of DNA were used for PCR. DNA from non-precipitated chromatin samples was extracted and used as input for PCR. Controls included isotype-matched IgG or Mock reactions which included all reagents except chromatin.

### Indirect immunofluorescence

Cells were grown on glass coverslips to 30 % confluence, rinsed 2X with PBS (phosphate-buffered saline, pH 7.4), fixed in 3 % paraformaldehyde for 15 min at 4 °C and rinsed twice with PBS. Fixed cells were permeabilized with 0.1 % Triton-X100 for 5 min, blocked in 2 % dry milk for 15 min, rinsed with PBS twice and incubated with primary antibody (anti-Mi2-β, ABCAM, MA) in PBS containing 1 % dry milk and 0.1 % Triton-X100 overnight. Secondary antibody, mouse mAB-Alexa-488 conjugate, was incubated for 1 h at room temperature. Cells were washed 3X with PBS and incubated in 1 μg/ml Hoescht nuclear dye for 20 min and then washed twice. Cells were visualized in a CARL ZEISS AXIOVERT 200 inverted microscope at a magnification of 63X.

### Protein immunoprecipitation

Cells were lysed with buffer and centrifuged at 4000 rpm for 2 min. Immunoprecipitation using anti-Mi-2β and anti-RbAp46 were completed as described previously [4].

### RNA interference assays

Cells were transfected with an EGFP-tagged L1RP vector. After the disappearance of EGFP fluorescence by epigenetic silencing, cells were replated and treated at 30-50 % confluence with 4 nM siRNA for 48 h followed by direct measurements of fluorescence.

### Chemical treatments

Cells were challenged with 3 μM BaP or 0.06 % dimethyl sulfoxide (DMSO) as vehicle control for 16 h to reactivate L1.

### Ethics statement

No ethics approval was required for the experimental work performed in this study.

## Results

### The NuRD corepressor complex is recruited to the L1 promoter

**Fig. 1:**
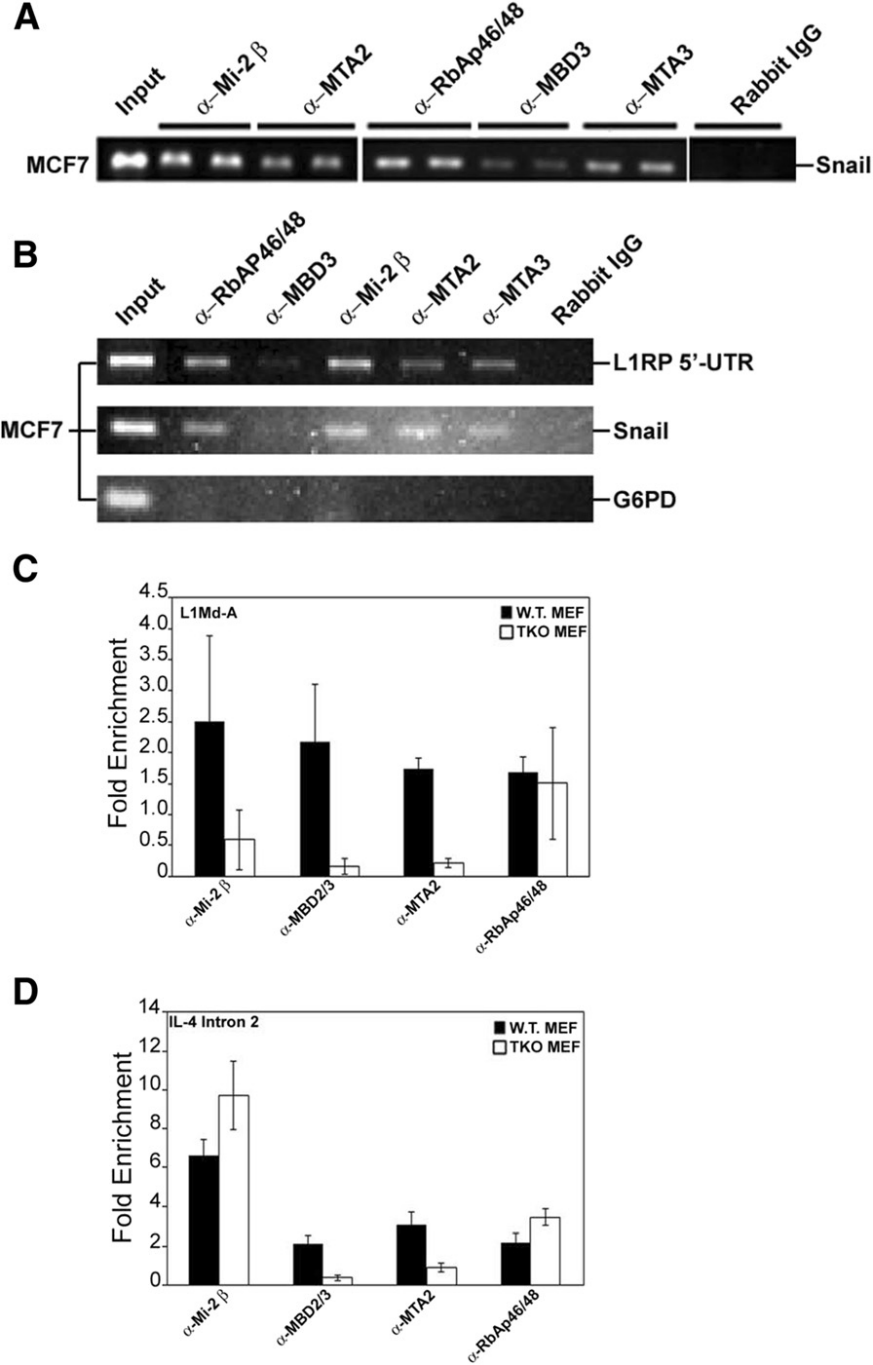
The NuRD co-repressor interacts with the human and murine L1 promoters in vivo. MCF7 breast cancer cells and early passage MEF cells were grown to 90 % confluence, crosslinked with DTBP and then treated with 1 % formaldehyde. Solubilized chromatin was immunoprecipitated using selected antibodies and amplified for the known MeCP1 target promoter Snail **a**, the human L1 promoter **b**, the murine L1Md-A type promoter **c** and the human IL-4 intron 2 transcriptional enhancer (**d**). Panel (**a**) shows two replicates for each for Mi2-β, MTA2, RbAp46/48, MBD3, MTA3 and IgG. Panel (**c**) shows the average of three separate chromatin immunoprecipitation assays for NuRD constituent proteins in WT and RB null MEFs (TKO). PCR products were separated in a 1 % agarose gel and visualized using a KODAK imager U.V. station. The results of three different experiments established the interaction of NuRD constituent proteins with the L1 promoter and implicated RB proteins in this process. Statistical differences for non-parametric data were evaluated using the Mann–Whitney test

The NuRD multiprotein complex includes the dermatomyositis-specific Mi2 autoantigen (Mi2-β), RB-associated proteins 46 and 48 (RbAp46 and RbAp48), MBD2 and MBD3, and metastasis-associated proteins 2 and 3 (MTA2 and MTA3) [9–11]. Validation trials were completed using soluble chromatin of human MCF7 cells and antisera against components of the NuRD corepressor complex compared to rabbit IgG as a control for non-specific interactions. Snail, a master switch for epithelial-to-mesenchymal transition in breast cancer that is regulated by the Mi2-β/NuRD complex [12], was used as a positive control in these experiments. As expected, specific enrichment for NuRD proteins on the Snail promoter was observed (Fig. 1a). PCR amplification of the human L1^RP^ 5′-UTR was compared to Snail and glucose 6-phosphate dehydrogenase (G6PD) gene promoters as positive and negative control sequences, respectively. All NuRD subunits specifically enriched the sample for the human L1RP 5′-UTR DNA sequence, but not the G6PD sequence (Fig. 1b). The presence of MBD3, MTA2 and MTA3 readily identified the complex as the MeCP1 multiprotein complex. The NuRD complex is involved in remodeling of nucleosomal particles to mediate formation of heterochromatin and localized domains of repressive chromatin. Mi2-β functions as an ATP-dependent helicase subunit with nucleosome remodeling activity [13], while RbAp46 functions as an adaptor subunit that recruits HDAC and inhibits HDAC-independent transcription [14, 15]. MBD3 may serve as a scaffold for assembly of the multimeric complex [16], while MTA2 and MTA3 aid in the recruitment of HDACs1 and 2 [16].

Subsequent experiments were conducted to examine the role of RB proteins in NuRD complex recruitment comparing wild type MEFs to TKO MEFs in which all three member of the RB family were genetically ablated [5]. In these studies, we tested antisera against Mi2-β, MBD3, MTA-2, and RbAp46/48, as well as histone H4 and a control rabbit serum. Specific enrichment for L1 5′-UTR was seen for Mi2-β, MBD2/3, MTA-2, and RbAp46 in wild type MEFs (Fig. 1c). Immunoprecipitation with total histone H4 antisera, but not control IgG yielded high levels of L1 DNA enrichment (not shown). In sharp contrast, markedly reduced enrichment was seen when chromatin from TKO MEFs was immunoprecipitated with serum against Mi2-β, MBD2/3, or MTA-2 (Fig. 1c). This pattern of response was specific to L1 sequences, as evidenced by markedly different profiles of NuRD protein recruitment to IL-4 intron 2 sequences (Fig. 1d). A comparison with IL-4 intron 2 sequences is pertinent given that genetic ablation of MTA-2 correlates with hyperinduction of IL-4 and abnormal T-cell activation in mice [17], and this response is partly mediated by a cis-acting element located in the second intron of the murine IL-4 gene [18]. As such, promoter specific patterns of RB-mediated regulation of L1 were identified. Together, these findings indicate that both human and murine L1 retroelements target the NuRD complex to their promoter regions, with RB proteins likely serving as stabilizers of macromolecular interactions.

### Silencing of NuRD subunits reactivates L1

**Fig. 2:**
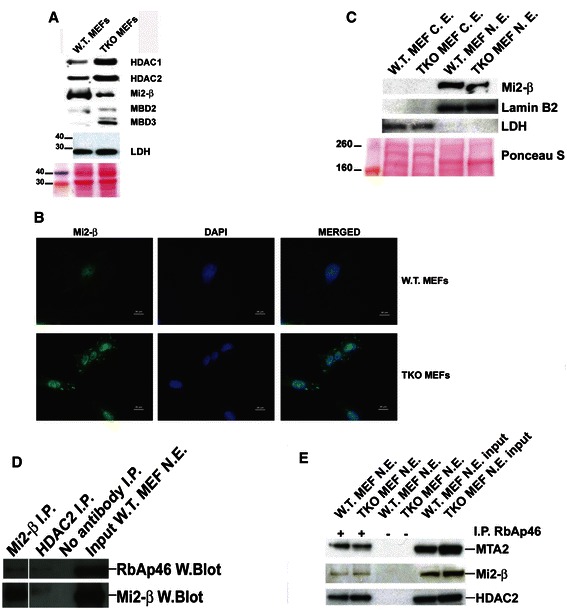
Recruitment of the NuRD corepressor complex in MEFs is influenced by RB proteins. **a** Wild type and TKO MEFs were grown to 90 % confluence, trypsinized and lysed using RIPA buffer supplemented with protease inhibitors. 20 μg of total protein was separated on a 4-12 % gradient PAGE gel and probed with antisera against selected targets. HRP-linked secondary antibodies were used for protein detection. Film exposure times varied from 30 s to 3 min. The abundance of Mi2-β was dramatically reduced in MEFs lacking RB proteins, while other members of the NuRD corepressor complex including, HDAC1, HDAC2 and MBD3, were increased. No differences in LDH levels and Ponceau staining as loading controls were observed. These results are representative of three independent experiments. **b** Immunofluorescence staining for Mi2-β and DAPI in WT and TKO MEFs. The signal in WT MEFs was confined to the nucleus, while that in TKO MEFs was distributed throughout the cell thus giving rise to lighter fluorescence signals. **c** Measurement of Mi2-b levels by Western blot analysis in nuclear extracts (NE) and cytosolic extracts (CE) of wild type and TKO MEFs. Lamin B2 and LDH were used as markers of purity for the nuclear and cytosolic extracts, respectively. Ponceau staining was used as a loading control for each of the fractions. While decreased nuclear Mi2-β levels as evidenced by Western blot analysis of nuclear protein in TKO cells compared to wildtype counterparts was observed, no signal was detected in the cytoplasmic fraction. **d** Immunoprecipitation of RbAp46 using anti-Mi2-β antibody in NE of WT MEFs. **e** Immunoprecipitation of MTA2, Mi2-β, and HDAC2 using anti-RbAp46 antibody in NE of WT and TKO MEFs

Since basal L1 mRNA levels are markedly upregulated in TKO MEFs [4], we sought to compare the relative abundance of NuRD corepressor proteins in wild type and TKO MEFs. HDACs1 and 2 were overexpressed in TKO MEFs, a finding consistent with previous reports from the laboratory [4]. Mi2-β was expressed at higher levels in wild type MEFs compared to RB null cells, while both MBD2 and MBD3 were enhanced in TKO MEFs compared to wild type MEFs. These findings suggest that the stoichiometry of the NuRD protein complex is unbalanced in the absence of RB, a hypothesis consistent with previous work showing that a reduction in Mi2 is paramount to histone-regulated nucleosome rearrangements [19]. Mi2-β was detected almost exclusively in the nucleus of WT MEF cells, while TKO MEFs showed Mi2-β localization in both the nuclear and cytoplasmic compartments, with punctate signal readily detected near the periphery of the cells (Fig. 2b). Relative decreases in nuclear localization of Mi2-β were confirmed by Western blotting using fractionated cytosolic and nuclear extracts from wild type and TKO MEFs (Fig. 2c). No signal was detected in the cytosolic fraction of either wild type or TKO MEFs, a finding consistent with the observation that delocalized Mi2-β associates with the plasma membrane (Fig. 2b). Immunoprecipitation of NuRD protein components using antibodies against Mi2-β (Fig. 2e) or RbAp46 (Fig. 2e) confirmed critical protein-protein interactions.

**Fig. 3:**
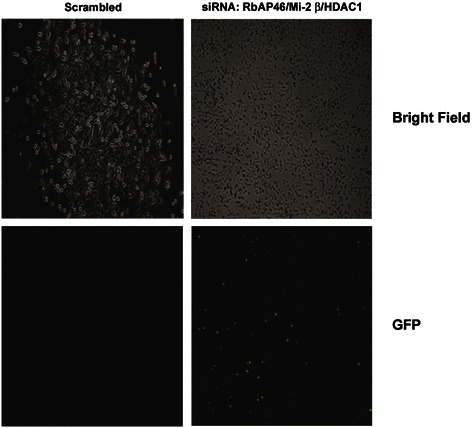
siRNA targeting of NuRD subunits reactivates ectopic L1. HeLa cells were transfected with a synthetic human L1 retroelement cloned into a pGL4.13 backbone vector where L1 ORF1p was tagged with EGFP fluorescent protein as a marker of reactivation. Following transfection, cells were grown for 7 days until no fluorescence was detected due to epigenetic silencing. Cells were then transfected with a mixture of siRNAs targeting Mi2-β, RbAp46 and HDAC1 compared to a non-target scrambled sequence. Images were acquired 48 h post transfection. Each experiment was performed in triplicate and images shown representative of the respective fields

To test for functional changes in L1 regulation, siRNA silencing of NuRD subunit components was examined next. HeLa cells were transfected with a vector encoding the full length human L1^RP^ carrying a chimeric ORF1p-GFP fusion protein. The backbone vector, pGL4.13, lacked an episomal sequence and is known to undergo silencing upon integration, as evidenced by the loss of fluorescence (Fig. 3). Transfected cells were then treated with a mixture of siRNAs directed against target and non-target siRNAs and fluorescence measured after 48 h. In keeping with earlier findings, we selected Mi2-β due to its importance in nucleosomal displacement, RbAp46 for its interaction with the pocket domain of RB proteins and HDAC1 for its role in silencing of core histones. In these experiments, HeLa cells were seeded at low densities to account for long latencies during repeated transfection and the efficiency of genetic knockdown confirmed by Western blotting (not shown). Figure 3 shows that non-target siRNA treatment did not change the repressive state of ectopic L1, as evidenced by the continued absence of fluorescence. Targeting of Mi2-β, RbAP46, and HDAC1 reactivated ORF1p-GFP expression, as evidenced by the reappearance of fluorescence signal. Of interest was the finding that treatment of cells with target siRNAs caused dramatic changes in cellular morphology. These findings indicate that disruption of the NuRD complex is sufficient to induce L1 reactivation.

### RB deficiency leads to unregulated L1 retroelement reactivation

**Fig. 4:**
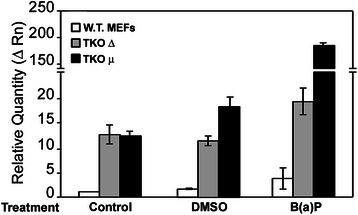
Genotoxic injury in the absence of the RB proteins leads to markedly upregulated L1 expression. Wild type MEFs and two independent TKO MEF clones were grown in 10 cm plates to 90 % confluence and treated with either medium alone, 0.06 % DMSO vehicle or 3 μM B(a)P for 16 h. Total RNA was extracted, quantified and cDNA prepared from 1 μg of starting material. 5 μL of a 1:20 dilution of the cDNA synthesis reaction were employed in qPCR studies with primers against β actin or murine L1 ORF1. Relative quantitative analyses were done using the Livak method of DDCt. Each experiment was performed at least three independent times

To examine the functionality of NuRD complexes in RB-deficient cells, we challenged wild type and TKO cells for 16 h with benzo(a)pyrene (BaP), a genotoxic carcinogen that reactivates L1 via epigenetic mechanisms [5]. BaP induces early enrichment of transcriptionally-active chromatin markers and reduces the association of DNA methyltransferase-1 (DNMT1) with the L1 promoter. These changes are followed by proteasome-dependent decreases in cellular DNMT1 and sustained reduction of cytosine methylation within the L1 promoter CpG island. Compared to DMSO controls, L1 expression increased in wild type MEFs challenged with the carcinogen (Fig 4), while two independent clones of TKO MEFs showed remarkable enhancement of the L1 response (Fig 4). Interestingly, the responsiveness of TKOμ was enhanced compared to its counterpart mutant line, but the origin of these differences in response have not been investigated. These data show that RB deficiency leads to unregulated L1 reactivation which combined with other findings indicate that L1 silencing in somatic cells is effected through interactions between RB family members and proteins within the NuRD macromolecular complex.

## Discussion

The NuRD complex represents a multiprotein complex that mediates formation of heterochromatin and localized domains of repressive chromatin. Its repressor activity involves assembly of a histone deacetylase macromolecular complex that couples histone modifications with nucleosome-stimulated ATPase activity. Mi2-β, RbAp46, MBD3, MTA 2 and MTA3 have been identified as critical NuRD multiprotein complex components. Mi2-β functions as an ATP-dependent helicase [13], RbAp46 recruits HDAC and inhibits HDAC-independent transcription [14, 15], MBD3 serves as a scaffold for complex assembly [16] and MTAs cooperate in the recruitment of HDACs [16].

The epigenetic silencing profile executed by the NuRD complex includes histone deacetylation, histone H3 lysine K9, H3 lysine 27, and H4 lysine 20 trimethylation, and DNA methylation, which together contribute to the recruitment of non-coding RNAs and repressors and corepressors to induce facultative or constitutive heterochromatin formation [20]. In this context, it is important to note that the polycomb repressive complex 2 (PRC2) mediates H3K27 methylation, which in turn facilitates PRC1 binding to methylated H3K27 and PRC1-dependent chromatin compaction [21]. Genes silenced during development are characterized by the presence of broad domains of repressive chromatin containing high levels of trimethylated H3K27 [20] or trimethylated H3K4 [22]. Repetitive elements such as L1 are rich in H3K9, H3K27, and H4K20 methylation and, importantly, their methylation density defines states of cellular differentiation [23]. Thus, repression of L1 activity is likely a conserved biological mechanism to ensure maintenance of the differentiated state.

Repetitive L1 sequences in the mammalian genome are extensively silenced via DNA methylation, and L1 methylation status is frequently used as a proxy of global cellular methylation [24, 25]. This laboratory has previously shown that BaP carcinogenesis is associated with L1 reactivation via mechanisms that involve E2F/RB-regulated opening of chromatin. Molecular reactivation is mediated by enrichment of H3K4Me3 and H3K9Ac, increased histone H3 acetylation at or near the 5′UTR and inhibition of DNMT1 recruitment and activity within the L1 promoter [4, 5]. Decreased DNMT1 is associated with decreased methylation at several CpG loci within the L1 promoter to mediate sustained retroelement reactivation. We also discovered that the Human Papilloma Virus (HPV) oncoprotein E7 associates with RB to differentially regulate L1 promoter activity in mammalian cells [26].

Our previous findings are consistent with the new data showing that L1 silencing by RB proteins is effected through the NuRD multiprotein complex. All major components of the NuRD complex are recruited to the silenced L1 promoter, and recruitment exhibits promoter-specificity. RB appears to orchestrate the recruitment of NuRD proteins and loss of RB function is associated with delocalization of Mi2-β from the nuclear to the cytosolic compartment. A critical role for RB in regulation of L1 retrotransposon was established in studies showing that genetic ablation of RB family members is associated with highly unregulated overexpression of L1 in MEFs. Recent studies using non-transformed human bronchial epithelial cells have shown that L1 reactivation by BaP is associated with disruption of NuRD complex assembly and function (Bojang et al. LINE 1 reactvation in human bronchial ephitelial cells requires dissambly of NuRD multiplrotein complex and loss or Mi2β and MBD2/3 correpressor functions, Submitted). Similar studies using cancer cell lines are confounded by differences in RB mutant status and RB-related functions. Such differences notwithstanding, comparative analyses of constitutive L1 expression have shown that human cells exhibit remarkably lower expression than murine cells [27], a finding consistent with species differences in rates of L1 retrotransposition. Importantly, L1 expression is considerably reduced in non-transformed human cells compared to transformed cells with dysregulated RB function. In keeping with these findings, Belancio et al. [28] showed that expression of full length and processed transcripts of L1 in normal human tissues, except possibly testis, is lower compared to transformed human cell lines. Direct comparisons of transformed cell lines showed that HeLa cells express lower levels of full-length L1 than MCF7 breast cancer cells, but that total L1-related products were comparable. The ability of transformed cell lines to support higher rates of L1 transcription, mRNA processing and retrotransposition is consistent with documented changes in RB regulation and function during the course of malignant transformation. In accord with these observations, normal human bronchial epithelial cells carrying wildtype RB exhibit low L1 expression, with high L1 inducibility upon genetic ablation of Mi2-β (Bojang et al. LINE 1 reactvation in human bronchial ephitelial cells requires dissambly of NuRD multiplrotein complex and loss or Mi2β and MBD2/3 correpressor functions, Submitted).

Collectively, our findings identify RB as a critical regulator of the NuRD multiprotein repressor complex assembly within the regulatory region of L1. NuRD recruitment is in turn responsible for effective silencing of L1 transcriptional activity. Genetic ablation of RB proteins destabilizes protein-protein interactions within the NuRD macromolecular complex to mediate nuclear rearrangement and delocalization of Mi2-β from the nuclear to cytoplasmic compartment. These findings suggest that Mi2-β is critical to transcriptional repression of L1. These findings are highly relevant to our understanding of oncogenesis and malignant progression given that NuRD is a key determinant of cellular differentiation, and that inappropriate recruitment of NuRD to specific loci contributes to tumorigenesis [29]. Given that L1 promoter hypomethylation has been associated with cancers at multiple sites [30], it is possible that NuRD-mediated oncogenesis is associated with disruption of transcriptional control of L1 retroelements. Simple inferences cannot be established given that the pathology of altered L1 expression in cancer involves genetic and epigenetic deficits associated with mutational L1 insertions [31] and unregulated profiles of gene expression [32].

## Conclusions

Our findings indicate that the L1 silencing by RB is effected through the NuRD macromolecular complex and implicate these molecular interactions in the formation of L1 heterochromatin structures. Full-length L1s in the mammalian genome are therefore potential targets for NuRD-mediated silencing and recruitment of silencing proteins such as the polycomb family of proteins. As such, unregulated silencing of L1 may play a central role in the initiation and progression of cancer.

## Abbreviations

DNMT: DNA methyltransferase
HAT: Histone acetyl transferase
HDAC: Histone deacetylase
HMT: Histone methyltransferase
LINE-1: Long interspersed nuclear element-1
MEF: Mouse embryo fibroblast
NuRD: Nucleosomal and remodeling deacetylase complex
RB: Retinoblastoma
TKO MEF: Rb protein family null mouse embryo fibroblast
